# Dexmedetomidine as an Adjuvant in Femoral Nerve Block for Ease of Preoperative Positioning and Postoperative Analgesia in Hip and Proximal Femur Surgery

**DOI:** 10.5812/aapm-170873

**Published:** 2026-04-30

**Authors:** Anushka Choudhary, Shaila Surendra Kamath, Priyanka Mahanta

**Affiliations:** 1 Department of Anaesthesiology, Kasturba Medical College Mangalore, Manipal Academy of Higher Education Manipal India

**Keywords:** Dexmedetomidine, Analgesia, Femoral Nerve Block, Bupivacaine

## Abstract

**Background:**

Perineural dexmedetomidine has been shown to improve block quality, accelerate onset, and prolong block duration when used as an adjuvant to local anaesthetics.

**Objectives:**

This study assessed the effects of adding dexmedetomidine to bupivacaine in an ultrasound-guided femoral nerve block (USG-FNB) on positioning for spinal anaesthesia and postoperative analgesia in patients undergoing surgery for hip and proximal femur fractures.

**Methods:**

This hospital-based observational study included 100 adults undergoing surgery for hip or proximal femur fractures. Using convenience sampling, participants were classified into two groups of 50 according to the femoral nerve block received: Group C received bupivacaine alone, and Group D received bupivacaine with dexmedetomidine at 1 µg/kg as an adjuvant. Once the visual analogue scale (VAS) score was < 3, patients were placed in the sitting position, and spinal anaesthesia was administered.

**Results:**

Median heart rate was significantly higher in Group D than in Group C at all assessed time points, except at 3 hours, when the groups were comparable. Participants in Group D achieved a VAS score < 3 significantly sooner after the block and had a significantly longer time to first rescue analgesia postoperatively than those in Group C (P = 0.001). Time to recovery from spinal anaesthesia was similar between groups. The mean number of rescue analgesic doses was significantly higher in Group C than in Group D.

**Conclusions:**

Dexmedetomidine demonstrated a superior analgesic profile as an adjuvant to a femoral nerve block, facilitating patient positioning for spinal anaesthesia and reducing postoperative rescue analgesic requirements.

## 1. Background

Osteoporosis is a major health issue, particularly among older adults, and is associated with fragility fractures of the hip, spine, and wrist. The increased risk of hip fracture in older adults is largely attributable to decreased bone mineral density and an increased incidence of falls (1). Appropriate analgesia has been shown to reduce the risks of delirium, prolonged hospital stay, and hospital-acquired complications (2, 3). Hip surgery is commonly performed under combined spinal-epidural anaesthesia (CSEA), which combines the benefits of spinal and epidural anaesthesia and is associated with fewer respiratory and circulatory complications (4, 5).

Ultrasound-guided femoral nerve block (USG-FNB) effectively reduces pain in femoral neck fractures; however, when a local anaesthetic is used alone, the duration of postoperative analgesia may be limited (6, 7). Several adjuvants, including fentanyl, clonidine, ephedrine, dexmedetomidine, and dexamethasone, have been studied as adjuncts to local anaesthetics to prolong analgesia. Perineural dexmedetomidine has been shown to improve block quality, accelerate onset, and prolong block duration (6, 7).

In patients undergoing lower-limb orthopaedic surgery, dexmedetomidine- and ropivacaine-assisted CSEA has been reported to reduce anaesthesia onset time, maintain perioperative haemodynamic stability, inhibit the release of pain mediators, and reduce cognitive dysfunction and delirium (8). Although dexmedetomidine has been evaluated as an adjuvant to local anaesthetics in femoral nerve blocks, evidence regarding its role in facilitating patient positioning for neuraxial anaesthesia in hip and proximal femur fracture surgery remains limited. The present study adds data on both preoperative benefits, specifically the time required to achieve adequate analgesia for spinal positioning, and postoperative analgesic outcomes of dexmedetomidine-enhanced USG-FNB in this population.

## 2. Objectives

This study evaluated the effect of USG-FNB using dexmedetomidine with bupivacaine on preoperative positioning for spinal anaesthesia and on postoperative analgesia in patients undergoing surgery for hip or proximal femur fractures. The objectives were to assess the time to onset of analgesia (visual analogue scale [VAS] score < 3) after femoral nerve block, the duration of postoperative analgesia, intraoperative haemodynamic parameters, and adverse effects.

## 3. Methods

### 3.1. Study Design and Setting

This hospital-based, comparative, nonblinded observational study was conducted over 2 years, from January 2023 through December 2024, at teaching hospitals affiliated with Kasturba Medical College Hospital, Mangalore, India. Approval was obtained from the Institutional Scientific Committee and Ethics Committee before study initiation, and the study was registered with the Clinical Trials Registry - India (CTRI) in accordance with institutional guidelines.

### 3.2. Sample Size and Study Participants

The sample size was calculated based on a previous study by Vinod et al. (9). With 80% power, an alpha error of 5%, and a 5% level of significance, the sample size was calculated using the following formula:

$$N=\frac{2{\left(Z_{1-\frac{\alpha }{2}}+Z_{1-\beta }\right)}^{2}S^{2}}{d^{2}}$$

A total of 100 patients (50 in each group) were enrolled using convenience sampling.

Adults aged 20 - 70 years with American Society of Anesthesiologists (ASA) physical status I or II who were scheduled for elective surgery for hip or proximal femur fractures under subarachnoid block, with an anticipated surgical duration of ≤ 3 hours, were included. Patients were excluded if they declined participation, had an ASA physical status of III or higher, required emergency surgery, had significant pulmonary, hepatic, cardiovascular, or renal disease, or were pregnant.

### 3.3. Methodology

Participants underwent a detailed preanaesthetic evaluation and routine investigations according to the surgical protocol. The procedure was explained in detail, and written informed consent was obtained. Participants were classified into groups according to whether dexmedetomidine was used as an additive to the femoral nerve block. Group C received USG-FNB with 25 mL of 0.25% bupivacaine plus 2 mL of normal saline. Group D received USG-FNB with 25 mL of 0.25% bupivacaine plus dexmedetomidine 1 µg/kg diluted to 2 mL with normal saline. USG-FNB was performed by an anaesthesia consultant with at least 3 years of experience, 30 minutes before spinal anaesthesia. The patients, anaesthesia consultants, and observers were not blinded at any time.

#### 3.3.1. Procedure

With the patient supine on the operating table, USG-FNB was performed under strict aseptic conditions using a linear HFL38 ultrasound probe with a bandwidth of 13 - 6 MHz and a depth of 6 cm (SonoSite Edge II; Fujifilm SonoSite Inc., Washington, United States). The femoral nerve was visualized, the skin and soft tissue were infiltrated with local anaesthetic, and a 22-gauge Stimuplex® Ultra 360® needle (B Braun) was advanced parallel to the long axis of the ultrasound beam. Once the needle was in proximity to the nerve, the study drugs were administered after negative aspiration. The VAS score was assessed, and once it was < 3, the patient was placed in the sitting position and spinal anaesthesia was administered with 15 mg of hyperbaric bupivacaine. The time required to reach a VAS score < 3 was recorded. Haemodynamic parameters, including heart rate, oxygen saturation, systolic blood pressure (SBP), diastolic blood pressure (DBP), and mean arterial pressure, were assessed at the time of the block, at the time of spinal anaesthesia, every 3 minutes for the first 15 minutes, every 15 minutes for 1 hour, and every 30 minutes thereafter until 3 hours. Adverse effects were recorded, including hypotension, defined as a 30% reduction from baseline SBP and treated with an intravenous bolus of ephedrine 0.1 mg/kg; bradycardia, defined as ≤ 50 beats/min and treated with intravenous atropine 0.02 mg/kg; hypertension, defined as a 30% increase from baseline SBP; and tachycardia, defined as a > 20% increase from baseline heart rate. Hypertension and tachycardia were treated with fentanyl 2 µg/kg.

Patients were monitored for recovery from spinal anaesthesia using the modified Bromage scale (10), the time to the first request for rescue analgesia, and the number of rescue analgesic doses required during the next 24 hours. Intravenous paracetamol 15 mg/kg was administered as rescue analgesia when the VAS score was > 3. The need for any additional analgesic was also recorded.

### 3.4. Statistical Analysis

Data were analysed using SPSS for Windows, version 26.0 (IBM Corp., Armonk, NY). Normality was assessed using the Shapiro-Wilk test. Continuous variables were compared using the Mann-Whitney U test or an unpaired t-test, as appropriate, and categorical variables were compared using the chi-square test. Results were presented in graphs and as variables. P < 0.05 was considered statistically significant.

## 4. Results

**Figure 1.**
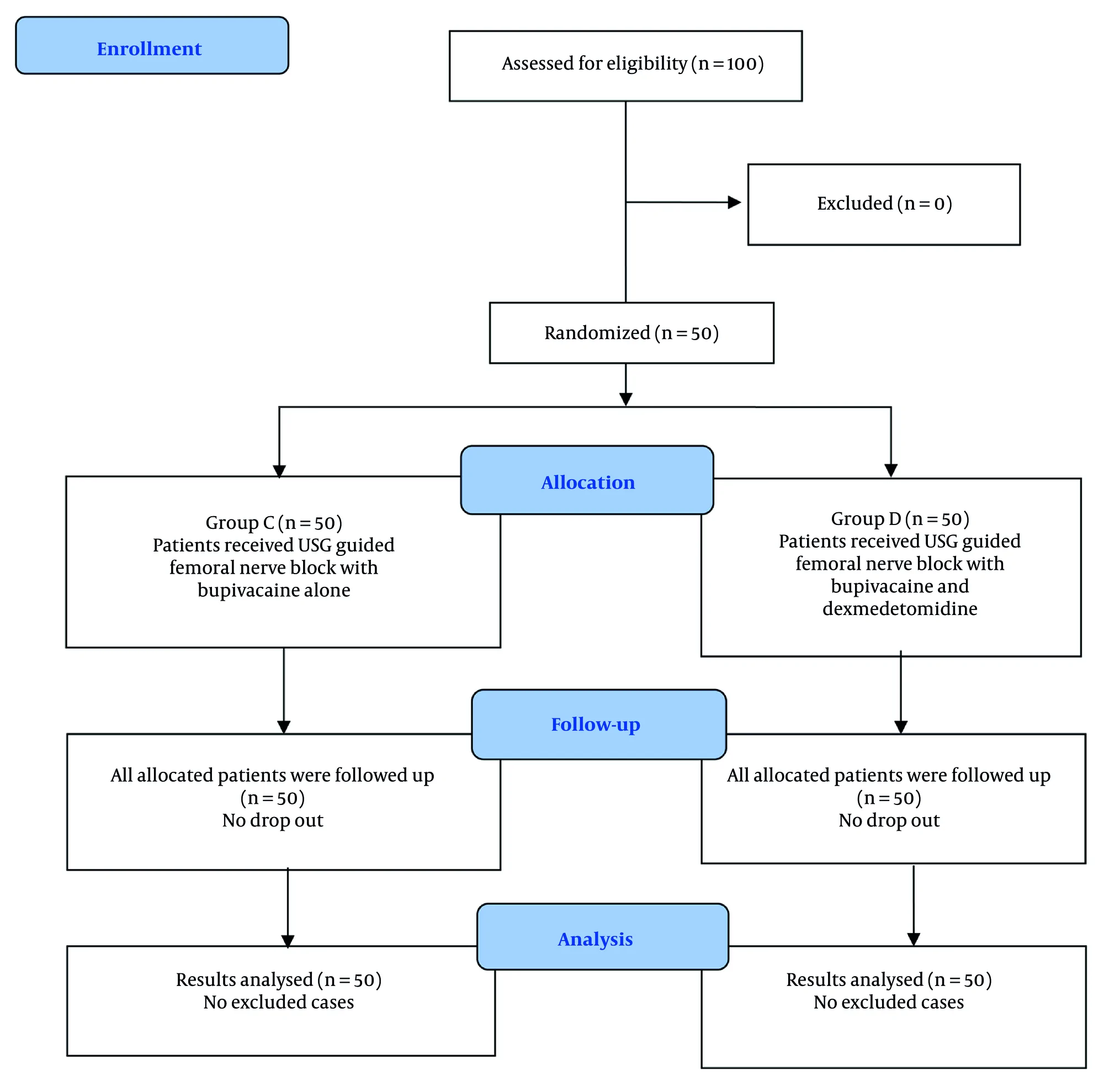
CONSORT flowchart of the enrolled patients

A total of 100 patients were included and classified into two groups of 50 according to the femoral nerve block received (Figure 1). Mean age, sex distribution, and type of surgery were comparable between the groups (P > 0.05) (Table 1). Median heart rate was significantly higher in Group D than in Group C at all assessed time points, except at 3 hours, when the groups were comparable (Table 2).

**Table 1.** Demographic Characteristics and Details of Surgery ^a^

Variables	Group C (n = 50)	Group D (n = 50)	P-Value
**Age (y)**	63.9 ± 3.6	64.2 ± 3.7	0.727
**Gender**			0.99
Male	22 (44)	21 (42)	
Female	28 (56)	29 (58)	
**Surgery**			0.84
Right PFN	26 (52)	24 (48)	
Left PFN	24 (48)	26 (52)	

^a^ Values are expressed as mean ± SD or No. (%). Abbreviation: PFN, Proximal femoral nailing.

**Table 2.** Comparison of Median Heart Rate (beats/min) Between Groups at Different Time Points ^a^

Times	Group C	Group D	Mann-Whitney U	P-Value
**Baseline**	75 (67.7 - 83.2)	82.5 (76.0 - 90.0)	2029	0.001 ^b^
**15 min**	88 (78 - 94.2)	99 (93 - 102)	1771	0.001 ^b^
**30 min**	85.5 (76 - 93.2)	96 (91.7 - 98)	1813.5	0.001 ^b^
**1 h**	82 (74.7 - 89.2)	87.5 (83.7 - 90.2)	2042.5	0.001 ^b^
**2 h**	76 (67.7 - 84)	82 (77 - 85.2)	2116	0.005 ^b^
**3 h**	75 (67.5 - 82)	76 (71 - 82)	2410	0.42

^a^ Values are expressed as median (IQR). Abbreviation: IQR, Interquartile range.

^b^ Statistically significant.

SBP was comparable between the groups through 2 hours; at 3 hours, SBP was significantly higher in Group D than in Group C (P = 0.038) (Table 3).

**Table 3.** Comparison of Median Systolic Blood Pressure (mm Hg) Between Groups at Different Time Points ^a^

Times	Group C	Group D	Mann-Whitney U	P-Value
**Baseline**	130 (120 - 138.5)	130 (124 - 138)	2411.5	0.433
**15 min**	122 (118 - 130)	122 (116 - 126)	2434	0.53
**30 min**	122 (117.5 - 128)	120 (116 - 124)	2263	0.07
**1 h**	122 (117.5 - 126)	122 (119.5 - 126.5)	2485.5	0.78
**2 h**	124 (120 - 128)	124 (120 - 128)	2503	0.87
**3 h**	122 (120 - 128)	126 (122 - 130)	2226.5	0.038 ^b^

^a^ Values are expressed as median (IQR). Abbreviation: IQR, Interquartile range.

^b^ Statistically significant.

Median DBP was comparable between the groups at baseline and at 1 and 2 hours. Differences were statistically significant at 15 minutes, 30 minutes, and 3 hours (Table 4).

**Table 4.** Comparison of Median Diastolic Blood Pressure (mm Hg) Between Groups at Different Time Points ^a^

Times	Group C, Median	Group D, Median	Mann-Whitney U	P-Value
**Baseline**	84 (78 - 88)	86 (81.5 - 90)	2259.5	0.066
**15 min**	74 (72 - 78.5)	72 (70 - 76)	2231	0.04 ^b^
**30 min**	74 (70 - 76.5)	71 (70 - 74)	2089	0.002 ^b^
**1 h**	76 (72 - 80)	74 (71.5 - 76)	2333.5	0.18
**2 h**	76 (72 - 80)	78 (74 - 80)	2519	0.9
**3 h**	76 (73.5 - 80)	80 (76 - 82)	2110	0.004 ^b^

^a^ Values are expressed as median (IQR). Abbreviation: IQR, Interquartile range.

^b^ Statistically significant.

Overall, participants in Group C had significantly higher oxygen saturation than those in Group D at all assessed time points (Table 5).

**Table 5.** Comparison of Median SpO2 (%) Between Groups at Different Time Points ^a^

Times	Group C	Group D	Mann-Whitney U	P-Value ^b^
**Baseline**	99 (98 - 100)	98 (98 - 99)	2020	0.001
**15 min**	99 (99 - 99)	98 (98 - 98)	1971	0.001
**30 min**	99 (99 - 99)	98 (98 - 99)	1867	0.001
**1 h**	99 (99 - 99)	98 (98 - 99)	1945	0.001
**2 h**	99 (99 - 99)	98 (98 - 99)	1767.5	0.001
**3 h**	99 (99 - 99)	98 (98 - 99)	1958.5	0.001

^a^ Values are expressed as median (IQR). Abbreviation: IQR, Interquartile range.

^b^ Statistically significant.

Participants in Group D reached a VAS score < 3 significantly sooner after femoral nerve block than those in Group C (P = 0.001) and had a significantly longer time to first postoperative rescue analgesia (P = 0.001). Six patients developed hypotension after spinal anaesthesia and received a 6 mg bolus of ephedrine. No patient required supplemental analgesia for positioning in the sitting position. The mean duration of surgery was 90 minutes, with a mean blood loss of 500 mL. Time to recovery from spinal anaesthesia was similar between the groups. The mean number of rescue analgesic doses was significantly higher in Group C than in Group D (Table 6).

**Table 6.** Comparison of Mean Time to VAS Score < 3, Recovery from Spinal Anaesthesia, Time to First Rescue Analgesia, and Number of Rescue Analgesic Doses Between Groups ^a^

Outcomes	Group C, Mean	Group D, Mean	t	P-Value
**Time to VAS score < 3 after block (min)**	11.30 ± 0.63	6.50 ± 0.54	40.9	0.001 ^b^
**Time to recovery from spinal anaesthesia (h)**	4.50 ± 0.50	4.60 ± 0.40	-1.005	0.3
**Time to first rescue analgesia (h)**	4.44 ± 0.50	9.34 ± 0.50	-47.9	0.001 ^b^
**Number of rescue analgesic doses**	3.62 ± 0.40	1.48 ± 0.50	21.5	0.001 ^b^

^a^ Values are expressed as mean ± SD.

^b^ Statistically significant.

## 5. Discussion

The present study evaluated dexmedetomidine as an adjuvant to bupivacaine for USG-FNB. Median heart rate was consistently higher in the dexmedetomidine group than in the control group at the assessed time points, although the difference was no longer significant at 3 hours. This finding contrasts with the well-documented pharmacodynamic profile of dexmedetomidine, which may produce bradycardia through activation of central α2-adrenoceptors, thereby reducing sympathetic tone and heart rate. Talke et al. and Bajwa et al. reported significant reductions in heart rate after dexmedetomidine administration (11, 12). The difference observed in the present study may reflect variability in individual autonomic responses, particularly under perioperative stress, which may transiently override the bradycardic effects of the drug. Procedural factors, such as patient anxiety or intraoperative stimuli during the initial assessment period, may also have contributed to sympathetic activation and the maintenance of a higher heart rate despite the pharmacological action of dexmedetomidine.

Median SBP was comparable between groups at baseline and through 2 hours. At 3 hours, Group D had a significantly higher median SBP than Group C (P = 0.038). Dexmedetomidine can produce transient changes in blood pressure through peripheral and central α2-adrenoceptor effects, and Talke et al. described transient hypertension during the initial phase of administration followed by a dose-dependent decrease in blood pressure (11). In the present study, however, no significant between-group SBP differences were observed before 3 hours in Table 3. The absence of sustained hypotension in the dexmedetomidine group may have been influenced by the dose, route, and speed of administration. Overall, the pattern is consistent with the haemodynamic stability described for dexmedetomidine as an adjuvant in regional anaesthesia by Marhofer et al. and Abdallah et al. (13, 14).

At baseline, median DBP was higher in the dexmedetomidine group, although the difference was not statistically significant (P = 0.06). At 3 hours, the dexmedetomidine group had a significantly higher DBP (P = 0.004). This pattern is consistent with previous findings suggesting that dexmedetomidine may initially increase arterial pressure through peripheral α2B-adrenoceptor-mediated vasoconstriction before centrally mediated sympatholytic effects predominate (11, 15). Marhofer et al. and Abdallah et al. also noted that the route of administration, dosage, and patient-specific factors can influence systolic and diastolic blood pressure responses (13, 14).

Participants in the dexmedetomidine group consistently had lower oxygen saturation, with statistically significant differences across the assessed time points. Baseline oxygen saturation was already significantly higher in the control group (P = 0.001), and this difference persisted throughout the observation period. However, no patient desaturated to the extent of requiring assisted ventilation. The lower oxygen saturation in the dexmedetomidine group may be related to its sedative effects. Patel et al. reported that dexmedetomidine has sedative properties while generally maintaining a safe respiratory profile and seldom causing significant hypoxia (16).

Participants in the dexmedetomidine group achieved a VAS score < 3 more rapidly (P = 0.001), suggesting that dexmedetomidine added to bupivacaine in femoral nerve block provided a faster onset of adequate analgesia. This finding is consistent with previous studies by Esmaoglu et al. and Marhofer et al. (17, 13). Singh et al. reported that dexmedetomidine as an adjuvant in the pericapsular nerve group (PENG) block was associated with prolonged analgesia, improved patient positioning, and lower postoperative pain than bupivacaine alone (18). Time to recovery from spinal anaesthesia was comparable between groups (P = 0.3), suggesting that adding dexmedetomidine to the peripheral nerve block did not adversely affect the regression of neuraxial blockade. This finding is consistent with Abdallah et al., who reported that perineural dexmedetomidine can prolong regional analgesia without significantly delaying motor or sensory recovery from spinal anaesthesia (14).

The dexmedetomidine group had a substantially longer time to first rescue analgesia than the control group (P = 0.001). In addition, the mean number of rescue analgesic doses was significantly higher in the control group (P = 0.001). These findings align with Brummett et al., who showed that dexmedetomidine combined with local anaesthetics can reduce postoperative analgesic requirements by extending the duration of peripheral nerve block (19).

### 5.1. Study Limitations

The primary limitation was the single-center design, which may restrict the generalizability of the findings to broader and more diverse populations. Although the sample size was adequate, a larger cohort might have improved the detection of subtle differences and rare adverse effects. The absence of blinding may have introduced selection, allocation, and measurement bias.

### 5.2. Conclusions

Dexmedetomidine demonstrated a superior analgesic profile as an adjuvant in femoral nerve block. It facilitated an earlier onset of effective pain control for positioning during spinal anaesthesia, delayed the need for rescue analgesia after spinal anaesthesia, and reduced the number of postoperative rescue analgesic doses without causing haemodynamic instability. These findings support the clinical utility of dexmedetomidine for improving patient comfort and postoperative pain management in lower-limb orthopaedic procedures.

## Data Availability

The dataset presented in the study is available on request from the corresponding author during submission or after publication.

## Clinical Trial Registration Code:

CTRI/2024/02/062223

## References

[1] Dhanwal DK, Dennison EM, Harvey NC, Cooper C (2011). Epidemiology of hip fracture: Worldwide geographic variation. Indian J Orthop.

[2] Shen Y, Liu W, Zhu Z, Liu S, Cao Y, Yan L (2023). Application of a preoperative pain management mode based on instant messaging software in elderly hip fracture patients: a randomized controlled trial. BMC Geriatr.

[3] McDonough CM, Harris-Hayes M, Kristensen MT, Overgaard JA, Herring TB, Kenny AM (2021). Physical Therapy Management of Older Adults With Hip Fracture: Clinical Practice Guidelines Linked to the International Classification of Functioning, Disability and Health From the Academy of Orthopaedic Physical Therapy and the Academy of Geriatric Physical Therapy of the American Physical Therapy Association. J Orthop Sports Phys Ther.

[4] Perlas A, Chan VWS, Beattie S (2016). Anaesthesia Technique and Mortality after Total Hip or Knee Arthroplasty. Anaesthesiology.

[5] Albanese AM, Ramazani N, Greene N, Bruse L (2022). Review of Postoperative Delirium in Geriatric Patients After Hip Fracture Treatment. Geriatr Orthop Surg Rehabil.

[6] Goel CP, Desai S (2021). Efficacy of dexmedetomidine as an adjuvant in femoral nerve block for post-op pain relief in hip surgery: A prospective randomized double-blind controlled study. J Anaesthesiol Clin Pharmacol.

[7] Packiasabapathy SK, Kashyap L, Arora MK, Batra RK, Mohan VK, Prasad G (2017). Effect of dexmedetomidine as an adjuvant to bupivacaine in femoral nerve block for perioperative analgesia in patients undergoing total knee replacement arthroplasty: A dose-response study. Saudi J Anaesth.

[8] Bai L, Zhao L, Jia F, Liu Y, Li P (2025). Effects of dexmedetomidine-ropivacaine assisted combined spinal-epidural anaesthesia on neutrophil-lymphocyte ratio and postoperative delirium in elderly patients with intertrochanteric femoral fracture. Frontiers in Pharmacology.

[9] Vinod M, Malashree G, Goud ES, Ravikumar K (2022). Dexmedetomidine as an Adjuvant to 0.25% Bupivacaine in Ultrasound Guided Femoral Nerve Block for Preoperative Positioning and Postoperative Analgesia in Patients Undergoing Elective Surgery for Fracture Shaft of Femur. Anesth Essays Res.

[10] Downie WW, Leatham PA, Rhind VM, Wright V, Branco JA, Anderson JA (1978). Studies with pain rating scales. Annals of the rheumatic diseases.

[11] Talke P, Chen R, Thomas B, Aggarwall A, Gottlieb A, Thorborg P (2000). The haemodynamic and adrenergic effects of perioperative dexmedetomidine infusion after vascular surgery. Anaesth Analg.

[12] Bajwa SJ, Arora V, Kaur J, Singh A, Parmar SS, Singh G (2011). Comparative evaluation of dexmedetomidine and fentanyl for epidural analgesia in lower limb orthopedic surgeries. Saudi J Anaesth.

[13] Marhofer D, Kettner SC, Marhofer P, Pils S, Weber M, Zeitlinger M (2013). Dexmedetomidine as an adjuvant to ropivacaine prolongs peripheral nerve block: a volunteer study. Br J Anaesth.

[14] Abdallah FW, Brull R (2013). Facilitatory effects of perineural dexmedetomidine on neuraxial and peripheral nerve block: A systematic review and meta-analysis. Br J Anaesth.

[15] Khan ZP, Ferguson CN, Jones RM (1999). Alpha-2 and imidazoline receptor agonists: Their pharmacology and therapeutic role. Anaesthesia.

[16] Patel R, Vadhadiya G, Gadani H, Gupta S (2023). Comparative Evaluation of Two Different Doses of Dexmedetomidine for Intra Operative Moderate Sedation During Spinal Anaesthesia. Archives of Anaesthesia and Critical Care.

[17] Esmaoglu A, Yegenoglu F, Akin A, Turk CY (2010). Dexmedetomidine added to levobupivacaine prolongs axillary brachial plexus block. Anaesth Analg.

[18] Singh R, Nazir N, Saxena A, Khanooja S, Kataria A, Panwar A (2026). (February 02, 2026) Efficacy and Safety of Dexmedetomidine as an Adjuvant to Bupivacaine in Ultrasound-Guided Pericapsular Nerve Group (PENG) Block for Proximal Femoral Fractures: A Randomised, Single-Centre Controlled Trial. Cureus.

[19] Brummett CM, Hong EK, Janda AM, Amodeo FS, Lydic R (2011). Perineural dexmedetomidine added to ropivacaine for sciatic nerve block in rats prolongs the duration of analgesia by blocking the hyperpolarization-activated cation current. Anaesthesiology.

